# Hypertriglyceridemia and Atherosclerosis: Using Human Research to Guide Mechanistic Studies in Animal Models

**DOI:** 10.3389/fendo.2020.00504

**Published:** 2020-08-06

**Authors:** Debapriya Basu, Karin E. Bornfeldt

**Affiliations:** ^1^Division of Endocrinology, Diabetes and Metabolism, New York University School of Medicine, New York, NY, United States; ^2^Department of Medicine, University of Washington Medicine Diabetes Institute, University of Washington School of Medicine, Seattle, WA, United States; ^3^Department of Pathology, University of Washington Medicine Diabetes Institute, University of Washington School of Medicine, Seattle, WA, United States

**Keywords:** angiopoietin-like 3, animal model, apolipoprotein, atherosclerosis, lipoprotein lipase, hypertriglyceridemia

## Abstract

Human studies support a strong association between hypertriglyceridemia and atherosclerotic cardiovascular disease (CVD). However, whether a causal relationship exists between hypertriglyceridemia and increased CVD risk is still unclear. One plausible explanation for the difficulty establishing a clear causal role for hypertriglyceridemia in CVD risk is that lipolysis products of triglyceride-rich lipoproteins (TRLs), rather than the TRLs themselves, are the likely mediators of increased CVD risk. This hypothesis is supported by studies of rare mutations in humans resulting in impaired clearance of such lipolysis products (remnant lipoprotein particles; RLPs). Several animal models of hypertriglyceridemia support this hypothesis and have provided additional mechanistic understanding. Mice deficient in lipoprotein lipase (LPL), the major vascular enzyme responsible for TRL lipolysis and generation of RLPs, or its endothelial anchor GPIHBP1, are severely hypertriglyceridemic but develop only minimal atherosclerosis as compared with animal models deficient in apolipoprotein (APO) E, which is required to clear TRLs and RLPs. Likewise, animal models convincingly show that increased clearance of TRLs and RLPs by LPL activation (achieved by inhibition of APOC3, ANGPTL3, or ANGPTL4 action, or increased APOA5) results in protection from atherosclerosis. Mechanistic studies suggest that RLPs are more atherogenic than large TRLs because they more readily enter the artery wall, and because they are enriched in cholesterol relative to triglycerides, which promotes pro-atherogenic effects in lesional cells. Other mechanistic studies show that hepatic receptors (LDLR and LRP1) and APOE are critical for RLP clearance. Thus, studies in animal models have provided additional mechanistic insight and generally agree with the hypothesis that RLPs derived from TRLs are highly atherogenic whereas hypertriglyceridemia due to accumulation of very large TRLs in plasma is not markedly atherogenic in the absence of TRL lipolysis products.

## Introduction

Whether elevated plasma triglycerides play a causal role in exacerbating cardiovascular disease (CVD) or represent a biomarker of CVD risk has been debated for 4 decades (1). In this comprehensive review organized in four major areas, we focus on how human research has guided mechanistic research in animal models of hypertriglyceridemia and potential effects of triglyceride (TG) modulation on atherosclerosis. These areas include defining (i) hypertriglyceridemia including origin and types of TG-rich lipoproteins (TRLs) and their remnants; (ii) current knowledge on association of hypertriglyceridemia with CVD risk in humans; (iii) animal models of hypertriglyceridemia and effects on atherosclerosis; and (iv) emerging strategies and drug targets to lower TGs and prevent atherosclerosis.

## What Is Hypertriglyceridemia?

Hypertriglyceridemia is often a biochemical diagnosis based on the levels of fasting plasma triglycerides (2). Normal fasting plasma TG levels are defined by current clinical guidelines as <1.69 mM (<150 mg/dL). Fasting TG levels of 1.69–2.25 mM (150–199 mg/dL) are considered moderately elevated, while fasting TGs above 2.26 or 2.83 mM (200 or 250 mg/dL) are considered high, and fasting TG levels above 5.65 mM (500 mg/dL) are considered severely elevated (2). An increased risk of acute pancreatitis is a consideration when TGs reach severely elevated levels (typically > 10 mM; close to and above 900 mg/dL) (3). Hypertriglyceridemia can be classified as primary types when a genetic susceptibility contribution is present and secondary types when no genetic component is detected (3). Secondary hypertriglyceridemia associates with poor diet, alcohol use, obesity, metabolic syndrome, and diabetes (2). Hypertriglyceridemia is often multifactorial, caused by genetic factors along with non-genetic factors favoring increased TRL secretion and/or reduced TRL clearance. Some genetic factors lead to hypertriglyceridemia only when associated with a second genetic or environmental factor, such as is the case for remnant removal disease (also known as type III hyperlipoproteinemia, broad band beta disease, or dysbetalipoproteinemia), which is most often caused by homozygosity of the *APOE2* genotype in combination with hormonal and/or environmental factors (4). Drugs like thiazides, beta-blockers, oral estrogens, tamoxifen, oral contraceptive pills, corticosteroids, atypical antipsychotics, isotretinoin, bile acid binding resins, anti-retroviral protease inhibitors, and immunosuppressive agents, such as sirolimus (mTOR inhibitors) also increase plasma TG levels (5).

**Figure 1:**
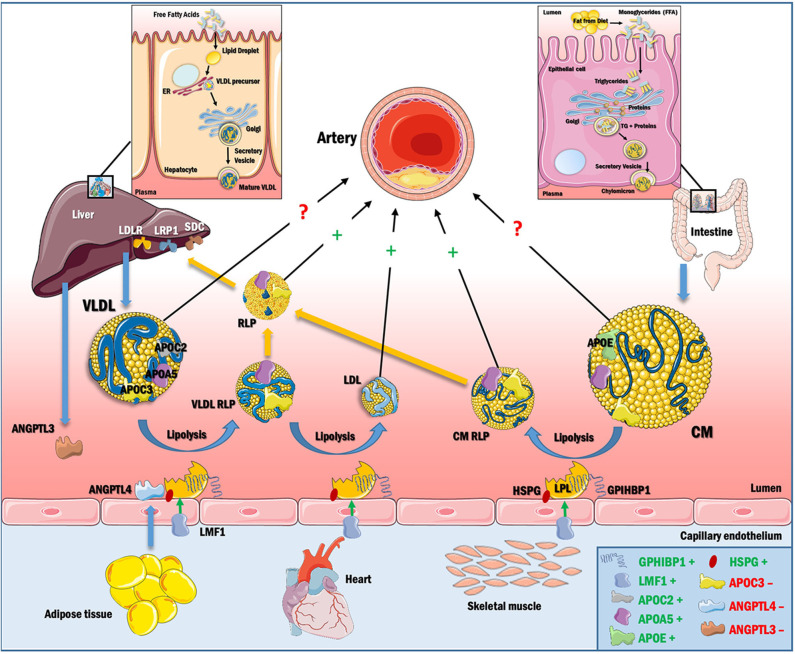
TRL synthesis and catabolism. Dietary TGs are absorbed and packaged as chylomicrons in the intestine, and secreted into the circulation via the lymphatic system. Chylomicrons contain APOB48 and acquire APOC2, APOC3, and APOE, some of which are transferred to other lipoproteins (primarily HDL) during lipolysis. LPL is synthesized in the parenchyma of heart, adipose and skeletal muscle and is further stabilized by LMF1. Active LPL remains anchored to GPIHBP1 and heparan sulfate proteoglycans (HSPGs). APOC2 and APOA5 activate LPL and help in chylomicron hydrolysis, releasing FFA for cellular energy requirements and cholesteryl ester-rich chylomicron remnants (RLPs), which are cleared by hepatic receptors in the LDL receptor family (LDLR and LRP1) with contributions from the HSPG syndecan (SDC) or are trapped in the artery wall. VLDL is synthesized in the liver using free fatty acid (FFA) from adipose tissue or from *de novo* lipogenesis and is then secreted into circulation. VLDL contains APOB100 on its surface as the major apolipoprotein. VLDL is hydrolyzed by LPL, creating VLDL RLPs, which accumulate in the artery wall or are further converted to LDL, the most atherogenic lipoprotein known. VLDL RLPs can also be removed by hepatic receptors or can be taken up by macrophages in arteries and other tissues. APOC3 reduces VLDL and chylomicron lipolysis by inhibiting LPL and by blocking TRL and RLP uptake by hepatic receptors. Hypertriglyceridemia thus results from increased production or decreased catabolism of chylomicrons and/or VLDL, and has a direct effect on the composition of LDL and HDL. RLPs are capable of penetrating the vascular endothelium and initiate the events of atherogenesis. The direct role of large VLDL and chylomicron in atherogenesis is however unclear, as it appears that these particles are too large to effectively enter the artery wall. In box, ^+^denotes positive regulation of LPL and ^−^denotes negative regulation of LPL.

TGs are present in circulation mainly in the cores of the TRLs—chylomicrons and very low-density lipoproteins (VLDL) and their remnant lipoprotein particles [RLPs, which include intermediate-density lipoproteins (IDLs) derived from VLDL]. Chylomicrons are synthesized and secreted from the intestine after a meal, whereas VLDLs are produced by the liver. Chylomicrons contain APOB48, a truncated form of APOB100, as the main apolipoprotein. Elevated TGs or hypertriglyceridemia can be due to greater intestinal and/or hepatic secretion of TRLs or reduced clearance of TRLs from the circulation (Figure 1). TRLs and their RLPs are enriched in apolipoprotein (APO) E, a ligand recognized by receptors of the low-density lipoprotein receptor (LDLR) family, including LDLR and the low-density lipoprotein receptor-related protein 1 (LRP1) receptor in the liver (6–8). While LDLR binds to, and participates in clearance of LDL by binding APOB100 (9), APOE-containing TRL and RLP particles are cleared through both LRP1 and LDLR in the liver.

A combination of human genetic approaches, including the study of genes that encode proteins identified by classical biochemistry, genes identified through genome-wide association studies (GWAS) of cohorts with a range of TGs, and genes identified by studies of families with TG phenotypes, have revealed that genetic factors contribute to a relatively large proportion of variation in plasma TG. In particular, homozygous or compound heterozygous mutations in genes that alter lipoprotein lipase (LPL) activity, like *LPL* itself*, APOC2, LMF1* (lipase maturation factor 1)*, GPIHBP1* (glycosylphosphatidylinositol anchored high density lipoprotein binding protein 1)*, APOA5*, and *GPD1* (encoding glycerol-3-phosphate dehydrogenase 1) are associated with high TG, although both severe forms of hypertriglyceridemia and moderate-to-mild hyperglycemia are usually of polygenic origin (10, 11). Bi-allelic pathogenic mutations in *LPL, APOC2, GPIHBP1, APOA5*, or *LMF1*, that lead to reduced LPL action, are used to confirm familial chylomicronemia syndrome, a rare autosomal recessive disorder characterized by severe elevation of TG levels, eruptive cutaneous xanthomas and acute pancreatitis (12). Furthermore, in some patients with chylomicronemia, autoantibodies against GPIHBP1 have been identified. These autoantibodies block the binding of LPL to GPIHBP1, leading to reduced LPL activity (13).

**Figure 2:**
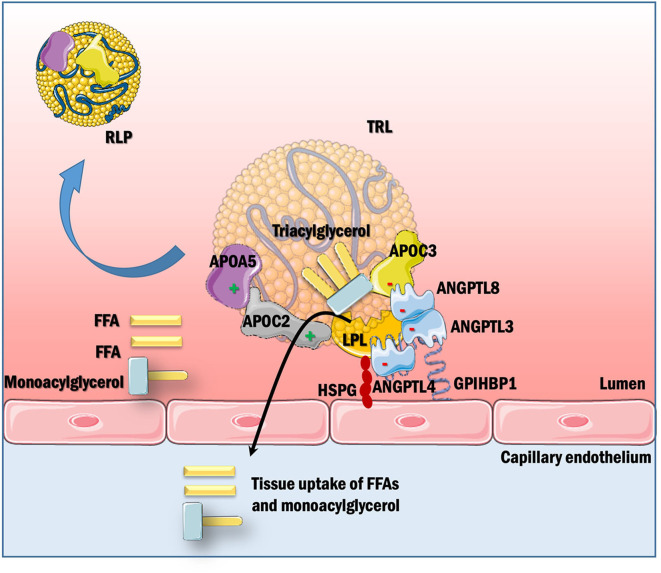
Posttranslational regulation of LPL-mediated TRL hydrolysis. LPL, synthesized in parenchymal cells of certain metabolic tissues, is secreted into the sub-endothelial space. GPIHBPI, expressed solely in capillary endothelial cells, is present on the basolateral side of endothelium where it captures LPL from the interstitial space and shuttles it across the endothelial cells to the luminal side of the capillary, which is the site of LPL action. This interaction with GPIHBP1 helps in TRL margination across the capillaries by enhancing lipolysis. LPL activity is further modulated by apolipoproteins within TRLs as well as secreted factors like angiopoietins. APOC2 is a vital cofactor for LPL activation, whereas APOC3 inhibits lipolysis and uptake of remnants in the liver. APOA5 acts to stabilize the LPL-APOC2 complex by helping TRLs to bind to the endothelial cell surface via HSPGs. ANGPTL3, ANGPTL4, and ANGPTL8 inhibit LPL depending on tissues and nutritional state. LPL hydrolyzes TRLs to generate free fatty acids (FFA) and monoacylglycerol, which are taken up by cells for their energy requirements. This process also generates RLPs, which contain a relative increase in cholesteryl ester and reduction in TGs as compared with their parent TRLs, and are considered to be highly atherogenic.

LPL is the major vascular enzyme catalyzing TG hydrolysis, generating free fatty acids and monoacylglycerol for tissue use, and RLPs. Hence, LPL is the principal player in the clearance of circulating TRLs. The binding of LPL to GPIHBP1 focuses the intravascular hydrolysis of TRLs on the surface of capillary endothelial cells in e.g., adipose tissue, skeletal muscle, and heart (14). LPL's action is modulated by activators like APOC2 and APOA5, inhibitors like APOC3 and angiopoietin-like proteins (ANGPTL3 and ANGPTL4), proper trafficking by LMF1 and anchoring by GPIHBP1, as shown schematically in Figure 2.

Additionally, receptor-mediated capture of RLPs produced after lipolysis by hepatic LDLR, LRP1 and syndecan 1 contributes to maintaining plasma TG levels (Figure 1).

## Hypertriglyceridemia Associates With Cardiovascular Disease Risk in Humans But Direct Evidence of Causality Is Scarce

Elevated plasma TGs have been known to associate with an increased risk of atherosclerotic CVD for decades. The first clues emerged over 40 years ago from the finding that patients with familial forms of hypertriglyceridemia exhibited an increased risk of coronary artery disease (15). Large epidemiological studies support the association between elevated TG levels and increased CVD risk (16–18), although the risk associated with elevated TGs is attenuated when adjusted for potential confounders, such as HDL-cholesterol (HDL-C) (19). Therefore, a causal link for this association has been challenging to determine, unlike increased LDL-cholesterol (LDL-C), which is a well-established causal risk factor for CVD. The difficulty determining a causal role for TGs in CVD in humans is in part due to the fact that plasma TG levels correlate negatively with HDL-C and can be present with high LDL-C, confounding its independent effect on CVD risk. Some studies however suggest association of elevated TG levels and CVD risk independent of LDL-C. For example, a recent study demonstrated that in older statin-treated subjects, elevated TG levels were associated with higher incidence of recurrent myocardial infarction in patients with LDL <100 mg/dL but not in those with LDL ≥100 mg/dL (20).

Weakening the case for a causal role of hypertriglyceridemia as a CVD risk factor are the clinical trials of TG lowering, which have mostly resulted in a lack of effect on CVD outcomes. Thus, fibrates and omega-3 fatty acids, which lower plasma TGs, have been mostly negative for CVD benefit (18), although subgroup analysis showed protective effects in subjects with high TGs and low HDL-C in some trials (18). So far, the only trials that produced a significantly beneficial effect on CVD outcomes were the REDUCE-IT (21) and JELIS (22) trials, in which subjects were treated with eicosapentaenoic acid (one of the omega-3 fatty acids). However, uncertainty exists regarding the mechanism of action of the eicosapentaenoic acid, as it has been suggested that the cardiobeneficial effects were likely due, at least in part, to mechanisms other than TG lowering (21). The negative results of the TG-lowering trials could have resulted from inclusion of individuals without hypertriglyceridemia in most of the fibrate trials, use of inadequate doses in most of the omega-3 fatty acid trials, or heterogeneity in the atherogenicity of the TG-carrying lipoproteins, at least in terms of TG as a marker of CVD risk (23). It is also possible that elevated TG levels are a general sign of the presence of a more atherogenic lipoprotein particle, such as the RLP. Because RLPs are lipolysis products of TRLs and therefore contain less TG/particle than their parent TRL, total plasma TG levels do not necessarily correlate with RLP levels (23).

Thus, although the association between hypertriglyceridemia and CVD risk in humans is strong, causal evidence is so far scarce. In fact, subjects with familial chylomicronemia syndrome, which is due to pathogenic mutations in genes controlling LPL itself or LPL activity, do not usually exhibit an elevated risk of atherosclerotic CVD despite the very high levels of TGs (12, 24). However, Mendelian randomization analyses indicate that TG-lowering LPL variants and LDL-C-lowering LDLR variants are associated with a similarly lower risk of coronary heart disease per unit difference in APOB (25). Thus, the relationship between plasma TG levels and atherosclerotic CVD is not straightforward, and high TG levels do not necessarily predict an increased CVD risk.

## What Are Remnant Lipoprotein Particles and Do They Promote Atherosclerosis?

The complex relationship between elevated TGs and atherosclerosis may be explained by a model in which increased atherosclerosis (and CVD) is not caused by the high TG content/lipoprotein particle, but to the presence of RLPs (chylomicron and VLDL lipolysis products), which are smaller than chylomicrons and VLDL and can more effectively exert pro-atherogenic effects in the artery wall (12). As described above, these RLPs are generated as LPL hydrolyzes TGs in the core of chylomicrons and VLDL, resulting in progressively smaller and TG-depleted particles with an increased ratio of cholesterol to TG. Although there is no universally agreed upon definition of RLPs, there is general agreement that these particles vary in size and composition. A slower clearance rate of RLPs, e.g., due to reduced LPL activity and incomplete conversion of TRLs to lipoproteins that have the greatest affinity for hepatic lipoprotein removal pathways, is thus predicted to result in a prolonged life-time of a spectrum of sizes of RLPs in circulation. These features have made it difficult to accurately quantify RLPs and assess their roles in CVD (23).

The concept that RLPs are highly atherogenic is supported by the finding that patients with remnant removal disease exhibit an increased risk of premature atherosclerotic CVD (26, 27). This condition is due to an impaired ability to clear RLPs through hepatic receptors of the LDLR family, which have a lower affinity for the APOE2 isoform of APOE (23). Because RLPs are larger than LDL, they contain 5–20 times more cholesterol per particle than does LDL (28). Once in the subendothelial space, RLPs are trapped by proteoglycans (18, 29) and can be taken up by lesional macrophages (30, 31). Furthermore, native unmodified RLPs promote rapid cholesterol accumulation in macrophages, in contrast to LDL, which has to be modified by e.g., oxidation to be taken up effectively by macrophages (30, 31). RLPs might therefore be more effective on a per particle basis than LDL at producing macrophage foam cells, a key cell type in atherosclerosis.

While these findings implicate RLPs in promoting atherosclerosis, their exact role in the atherogenic process is still unclear. However, the findings discussed above suggest that lower TG levels might be associated with an increased CVD risk, as compared with severe hypertriglyceridemia, if accompanied by elevated levels of more atherogenic RLPs derived from TRLs.

In order to tackle the issue of whether TRLs or RLPs are causatively involved in exacerbating atherosclerosis, animal models have been used to investigate potential atherogenic effects of TRLs and RLPs and to provide mechanistic insight through an approach that has sometimes been termed “reverse translation,” “human-first,” or “bedside-to-bench” research, which begins with observations in the clinic and works “backwards” to uncover the mechanisms behind the clinical observations (32). In fact, the first genetic mouse model of atherosclerosis with advanced lesions—the APOE-deficient mouse—was generated in the Breslow and Maeda laboratories in the early 1990's in part to mimic the impaired clearance of RLPs and severe premature coronary and peripheral vessel risk observed in patients with remnant removal disease (33, 34).

## What Have We Learned From Animal Models?

Like the human studies of subjects with different genetic alterations that are associated with changes in plasma TRLs and RLPs, studies in different animal models generally support the hypothesis that RLPs derived from TRLs are highly atherogenic whereas high TG levels due to accumulation of very large TRLs are much less atherogenic. Thus, the APOE-deficient (*Apoe*^−/−^) mouse exhibits a dramatic atherogenic phenotype concomitant with a lipoprotein profile enriched in VLDL and RLPs even when fed a low-fat diet (33), whereas LPL-deficient mice and GPIHBP1-deficient mice, which have very high TG levels due to lack of lipolysis and generation of RLPs exhibit delayed formation of small atherosclerotic lesions (35, 36). Likewise, high-cholesterol diet-fed rabbits support the concept that accumulation of RLPs relatively enriched in cholesterol and depleted of TGs lead to atherosclerosis whereas increased levels of large TRLs cause less atherogenesis (37–39).

The most common animal models of hypertriglyceridemia and the evidence emerging from each of these models related to atherosclerosis are discussed below and are summarized in Table 1. Further research using various animal models and mechanistic *ex vivo* studies is likely to shed important new light on mechanisms whereby RLPs promote atherosclerosis.

**Table 1:** Animal models of altered TG levels and effects on atherosclerosis.

	**Animal model**	**Likely mechanism of action**	**Role in atherosclerosis**
Mouse models	APOE-deficiency	Defective TRL and RLP clearance	Spontaneous atherosclerosis, severe with high-fat diets (33, 34)
	APOE*3-Leiden transgenic	Defective RLP clearance	Increased atherosclerosis (40, 41)
	Global LPL-deficiency	Reduced LPL activity	Small spontaneous lesions in old mice (35)
	Induced LPL-deficiency	Reduced LPL activity	No effect on lesion regression (42)
	LPL overexpression	Increased LPL activity	Reduced atherosclerosis (43, 44)
	GPIHBP1-deficiency	Reduced LPL activity	Small spontaneous lesions (36)
	LMF1-deficiency	Reduced LPL activity	Unknown
	APOC2-deficiency	Reduced LPL activity	Unknown
	APOC3-deficiency	Increased RLP clearance	Reduced atherosclerosis in diabetes model (45)
	APOC3 overexpression	Reduced LPL activity and hepatic clearance of TRLs and RLPs	Increased atherosclerosis (46, 47)
	APOA5-deficiency	Reduced LPL activity	Unknown
	APOA5 overexpression	Increased LPL activity	Reduced atherosclerosis (48)
	CREB-H-deficiency	Reduced LPL activity	Increased atherosclerosis (49)
	ANGPTL3-deficiency	Increased LPL and EL activity	Reduced atherosclerosis (50)
	ANGPTL4-deficiency	Increased LPL activity	Reduced atherosclerosis (51)
	Diabetes-induced	Reduced LPL activity and increased APOC3	Increased atherosclerosis (45, 52, 53)
Rat models	Corpulent gene (cp/cp); JCR:LA-cp	Increased VLDL secretion, saturation of LPL activity	Increased atherosclerosis (54)
	APOE-deficiency	Defective TRL and RLP clearance	Increased atherosclerosis (55–57)
	Sucrose/fructose diet induced	Increased *de novo* lipogenesis	Unknown
Rabbit models	Watanabe Heritable Hyperlipidemic (WHHL) model fed high-cholesterol diet	Reduced clearance of APOB-containing lipoproteins	Increased atherosclerosis (58, 59)
	Hereditary Postprandial Hypertriglyceridemic (PHT) model, standard diet	Increased postprandial lipemia	Early lesions (60)
	Thomas Hospital (STH) rabbit	Increased production of APOB in both the LDL and VLDL fractions	Increased atherosclerosis (61)
Pig models	Göttingen minipigs on a dietary intervention consisting of high-fat/high-energy diet	Delayed TG absorbance and clearance	Increased atherosclerosis (62)
	APOC3 transgenic	Delayed TRL clearance	Unknown
Primate models	Rhesus macaques, high-fructose diet	Increased *de novo* lipogenesis	Unknown
Zebrafish model	APOC2 loss-of-function mutant	Decreased LPL activity	Lipid laden macrophages in vasculature (63)

*References are shown in parentheses*.

## Animal Models of Hypertriglyceridemia and Their Susceptibility to Atherosclerosis

### Monogenic and Digenic Mouse Models of Hypertriglyceridemia

#### APOE-Deficiency

The *Apoe*^−/−^ mouse model has been extensively used as a model of atherosclerosis since its generation (33, 34). APOE, which is enriched in TRLs and RLPs, is recognized by the hepatic LRP1 and LDL receptors, leading to clearance of TRLs and RLPs (Figure 1). *Apoe*^−/−^ mice fed low-fat chow diet have lipoprotein profiles enriched in VLDL-cholesterol and RLP (IDL)-cholesterol when analyzed by fast phase liquid chromatography (33). The LDL receptor-deficient mouse (*Ldlr*^−/−^ mouse), which was generated as a model of homozygous familial hypercholesterolemia (caused by LDL receptor mutations in humans), on the other hand, exhibits an increase primarily in LDL-cholesterol when fed a chow diet, reflecting impaired clearance of LDL through hepatic LDL receptors (64). These *Ldlr*^−/−^ mice are largely free of atherosclerosis when fed a chow diet (64). The differences in severity of atherosclerosis phenotypes between chow-fed *Apoe*^−/−^ mice and *Ldlr*^−/−^ mice are most likely due to the elevated levels of TRLs and RLPs in *Apoe*^−/−^ mice, as compared with *Ldlr*^−/−^ mice, which in turn is due to the severely impaired hepatic clearance of APOE-containing TRLs and RLPs in *Apoe*^−/−^ mice. However, APOE also likely prevents atherosclerosis in part through its anti-inflammatory effects (65). Furthermore, the finding that mice deficient in both APOE and LDLR (*Apoe*^−/−^ *Ldlr*^−/−^ mice) exhibit higher levels of VLDL and chylomicron RLPs than do *Ldlr*^−/−^ mice, revealed that TRLs and RLPs are also cleared through a receptor (LRP1) distinct from the LDLR (64). The *Apoe*^−/−^ mouse also shows higher levels of APOB48 (the truncated form of APOB100 associated primarily with chylomicrons and their remnants) whereas the *Ldlr*^−/−^ mouse has primarily APOB100 in plasma (64). Thus, both forms of APOB are atherogenic. When fed a Western-style high-fat diet, *Apoe*^−/−^ mice develop markedly higher levels of VLDL and IDL, and increased atherosclerosis as compared to *Apoe*^−/−^ mice fed a chow diet (33).

As mentioned above, TRLs and RLPs are cleared by both the LDLR and LRP1 in liver. This conclusion is supported by the finding that inducible liver-specific LRP1-deficiency is sufficient to increase plasma chylomicron RLP levels, but only when the mice are also deficient in LDLR (7, 66, 67). Subsequent studies demonstrated that hepatic expression of LRP1 protects from atherosclerosis through additional mechanisms independent of clearance of APOE-containing RLPs because liver-targeted LRP1 deletion in *Apoe*^−/−^ *Ldlr*^−/−^ mice increased atherosclerosis (68).

Together, these studies show that loss of APOE leads to a dramatic increase in atherosclerosis in mouse models, an effect that appears to be mediated in large part by the increased plasma levels of TRLs and RLPs. Furthermore, mouse studies have shown that these APOE-containing TRLs are cleared from circulation through the hepatic receptors LDLR and LRP1.

#### APOE^*^3-Leiden Transgenic Mice

APOE has three major alleles; *APOE2, APOE3*, and *APOE4*. A mutant APOE3 allele that has a lower LDLR receptor affinity than wildtype APOE3 has been identified. This APOE^*^3-Leiden gene (^*^ denotes the mutant allele), which is associated with a dominantly inherited form of remnant removal disease was first described in a large Dutch family (69) and is very rare as compared with the *APOE2/APOE2* genotype more often associated with remnant removal disease (69). Transgenic mice harboring this mutant allele were generated by introducing a human APOE^*^3-Leiden gene construct isolated from the APOE^*^3-Leiden proband, consisting of the APOE^*^3-Leiden and *APOC1* genes and a promoter element that regulates the expression of the *APOE* and *APOC1* genes (70). These mice exhibited significantly elevated levels of total plasma cholesterol and TGs on a regular chow diet. When fed a cholesterol-rich diet, total plasma cholesterol and TG levels increased dramatically. This increase was observed mainly in the VLDL and LDL-sized particles (70). On a high fat/high cholesterol diet, the APOE^*^3-Leiden mice develop atherosclerotic lesions in the aortic arch, the descending aorta, and the carotid arteries, with characteristics of human vascular pathology, varying from fatty streaks to mild, moderate, and severe plaques (40). In another study, the effect of monocyte/macrophage-expression of APOE and APOE^*^3-Leiden was investigated by transplanting bone marrow into atherosclerosis-prone *Apoe*^−/−^ mice. APOE^*^3-Leiden transplanted bone marrow was less effective in reducing atherosclerosis, as compared with bone marrow cells expressing wildtype murine APOE (41). The APOE^*^3-Leiden transgenic mouse model is perhaps the most relevant model of impaired RLP removal and resulting increased atherosclerosis. However, the presence of the *APOC1* transgene makes data generated by this model as far as the contribution of the APOE3 mutation to atherosclerosis difficult to interpret because APOC1 aggravates atherosclerosis and increases plasma TGs and cholesterol (71), maybe in part through its ability to inhibit LPL activity (72) and reduce hepatic VLDL clearance (73).

An APOE^*^3-Leiden mouse model lacking the APOC1 region has also been generated (73). However, this mouse model needs further characterization in terms of atherosclerotic lesion development.

#### Lipoprotein Lipase-Deficiency

As discussed above, LPL is the primary rate-limiting enzyme for hydrolysis of TGs present in the core of chylomicrons, VLDL, and RLPs, generating free fatty acids and monoacylglycerol, which are taken up and used by extrahepatic tissues (Figure 2). Through lipolysis, LPL-mediated catabolism of TRLs in the bloodstream also generates progressively smaller and cholesterol-enriched (TG-depleted) atherogenic lipoprotein particles, such as chylomicron RLPs, VLDL RLPs, and LDL (74, 75). LPL is also vital for generation of HDL (76, 77). This is because as TGs in TRLs are lipolyzed, a significant proportion of the excess phospholipids and cholesterol of the shrinking TRL particle are transferred to HDL, together with APOC apolipoproteins (76) and because of reduced catabolism of APOA1 (the main structural protein of HDL) (78). Therefore, low LPL activity leads to hypertriglyceridemia and low HDL levels.

LPL is synthesized in the parenchymal cells of adipose tissue, skeletal muscle and heart, and is secreted into interstitial spaces, where it is captured by the capillary endothelial cell protein GPIHBP1, and transported to the capillary lumen (2). LPL is also synthesized by lesional macrophages (79). LPL is actively regulated by numerous proteins (Figure 2). Based on previous work, LPL has been assumed to be active as a homodimer, however, a recent study shows that that LPL and GPIHBP1-bound LPL can be active in a monomeric state (80).

Deficiency in LPL activity in humans (type I hyperlipoproteinemia or familial chylomicronemia syndrome) is associated with massive chylomicronemia, low HDL-C levels, and recurrent attacks of pancreatitis when not controlled by a strict diet (81). In contrast to humans, homozygous LPL-deficient mice do not survive suckling and die between 18 and 24 h after birth. Adenovirus-based transient expression of LPL during the suckling period was used in an effort to rescue these LPL-deficient mice. After a single intraperitoneal injection of LPL-expressing virus immediately after birth, more than 90% of the LPL-deficient pups survived the first day of life (82). Furthermore, 3% of these mice survived the entire suckling period and lived for up to 20 months, although LPL activity in mouse tissues and in plasma from mice injected with heparin to liberate LPL tethered to endothelial cells (postheparin plasma) was undetectable in all animals after 6 weeks of age. Adult LPL-deficient mice are smaller than their littermates until 2–3 months of age and exhibit very high TG levels in the fed (5,000 vs. 100 mg/dL in wildtype controls) and fasted state (2,000 vs. 70 mg/dL in wildtype controls). LPL-deficient mice differ from LPL-deficient humans in that they do not develop pancreatitis even when very high plasma TG levels are present.

Because of the drawbacks of the whole-body LPL-deficient mouse model discussed above, several tissue-selective LPL-deletion models and other approaches to reduce LPL expression have been used. For example, skeletal muscle specific- or endothelial cell-specific overexpression of an LPL minigene has been shown to rescue *Lpl*^−/−^ mice from neonatal lethality (83, 84). Heart-specific LPL expression to levels found in wildtype mice prevents lethality and leads to almost complete normalization of circulating TG levels in mice deficient in skeletal muscle and adipose tissue LPL (85) whereas cardiac-specific deletion of LPL leads to only mildly elevated plasma TGs (86). Whole-body inducible LPL-deficient mice have also been generated. These mice are viable when LPL deletion is induced in adult mice (87). On a chow diet, these mice develop high TGs (~500 mg/dL) and when fed a high-fat diet, TG levels can reach 3,000 mg/dL (67, 87).

Aged LPL-deficient mice (15 month-old) spontaneously develop early fatty streaks when fed a chow diet (35), suggesting that the high levels of TRLs in these mice can promote some atherosclerosis even in the absence of RLP generation, consistent with the hypothesis that large TRLs can be modestly atherogenic. Whole-body LPL-deficient mice have never been crossed to *Ldlr*^−/−^ or *Apoe*^−/−^ mice, so the phenotype is still unknown. Recently, mice with inducible LPL-deficiency have been used to study regression of lesions of atherosclerosis. These studies showed that near-complete loss of LPL caused elevated TG levels of ~500 mg/dL, but did not impede regression of atherosclerosis measured as lesional macrophage content (42), supporting the hypothesis that large TRLs in the absence of RLP generation are not markedly atherogenic. Consistently, heterozygous LPL-deficient mice crossed with *Ldlr*^−/−^ mice and fed a high-fat diet are hypertriglyceridemic, as compared with *Ldlr*^−/−^ controls but exhibit no increase in atherosclerosis progression (88). The authors suggested that the results could be explained by the loss of LPL in the vascular wall, which would prevent the action of pro-atherogenic lipolysis products, including atherogenic RLPs, locally in the lesion (89–91). Thus, macrophage-targeted deficiency of LPL in mice reduces atherosclerotic lesions (90, 91).

The direct relationship between LPL and atherosclerosis is complex. *Ldlr*^−/−^ mice as well as *Apoe*^−/−^ mice overexpressing LPL are resistant to diet-induced atherosclerosis, due to the efficient clearance of TRLs and RLPs in these mice (43, 44). Furthermore, it is probable that the RLPs are different in *Ldlr*^−/−^ mice vs. *Apoe*^−/−^ mice with normal LPL or supraphysiological levels of LPL. In *Ldlr*^−/−^ mice, which express APOE, the increased lipolysis due to LPL overexpression generates smaller, TG-poor and cholesterol-poor VLDL RLPs, which are cleared faster. In *Apoe*^−/−^ mice with LPL overexpression, VLDL remnant TG is lower but cholesterol content is unchanged. The exact reason for this difference is unknown. However, lack of APOE could interfere with RLP clearance. Although LPL overexpression is atheroprotective in both models, *Ldlr*^−/−^ mice with LPL overexpression exhibit suppressed lesion formation 10 times greater than *Apoe*^−/−^ mice with LPL overexpression. Another interesting perspective is that in *Apoe*^−/−^ mice with or without LPL overexpression, non-HDL cholesterol levels are similar, yet the atherosclerosis is suppressed. This model represents one mouse model showing that lowering TRL and RLP levels is atheroprotective, independent of cholesterol levels. A similar example of such a model is the diabetes model in which APOC3 has been silenced, discussed below. These findings are consistent with the notion that TRLs/RLPs are pro-atherogenic even without changes in LDL.

The results from LPL-deficient mice and mice with induced LPL-deficiency, which do not readily develop atherosclerosis or exhibit impaired lesion regression, appear to be consistent with studies on homozygous LPL-deficient human subjects, whereof very few develop premature atherosclerosis (92–94), supporting the hypothesis that homozygous LPL deficiency causes accumulation of large TRLs which cannot effectively promote atherosclerosis. On the other hand, heterozygous LPL-deficiency is associated with increased ischemic heart disease, elevated risk of coronary atherosclerosis and diminished clinical event–free survival (95, 96). This evidence supports a second hypothesis, which is not incompatible with the first hypothesis, that partial loss of LPL is pro-atherogenic, possibly because lipolysis of TRLs is less efficient than when LPL is normally expressed, causing RLPs to circulate for longer periods of time and accumulate in the artery wall.

The difficulty in interpreting the phenotype of LPL-deficiency and partial LPL-deficiency stems from the known role of LPL in reduction of overall TG levels through increased RLP levels and subsequent clearance of TRLs and RLPs. Therefore, complete LPL-deficiency leads to increased levels of very large TRLs and reduced atherogenic RLPs, whereas a partial LPL-deficiency is likely to result in increased levels of atherogenic RLPs with an increased retention time in circulation and increased accumulation in the artery wall, as compared to wild type levels of LPL. Moreover, LPL expressed in macrophages has detrimental effects on atherosclerosis, which are distinct from its effects on TRL levels. There is no animal model as of yet, in which LPL-generated RLPs can be altered independently of TRLs.

#### Glycosylphosphatidylinositol-Anchored HDL-Binding Protein 1 (GPIHBP1)-Deficiency

GPIHBP1 is a GPI-anchored protein expressed in the capillaries of heart, adipose tissue, and skeletal muscle (97–99) (Figure 1). GPIHBP1, present at the basolateral side of the endothelial cell, captures LPL from the subendothelial spaces and transports it across the endothelial cell to the capillary lumen (100, 101). GPIHBP1 is critically important for the margination (the partitioning of large TRLs along the capillary endothelium) of lipoproteins by facilitating TRL hydrolysis by LPL (102). In addition, GPIHBP1 stabilizes the structure and catalytic activity of LPL (103, 104). Recently, GPIHBP1 proved to be crucial for solving the structure of LPL (105, 106).

Mice lacking *Gpihbp1* have severe chylomicronemia due to a decreased ability of LPL to efficiently metabolize TRLs (97). Nearly all of the cholesterol and TGs in plasma of *Gpihbp1*^−/−^ mice are associated with large TRLs 50–135 nm in diameter, leading to plasma TG levels close to 3,000 mg/dL, and both male and female *Gpihbp1*^−/−^ mice spontaneously develop small atherosclerotic lesions in the aortic root when fed a chow diet (36), phenocopying the spontaneous atherosclerosis in chow-fed *Lpl*^−/−^ mice (35). These lesions are much smaller than those that develop in *Apoe*^−/−^ mice, which have an accumulation of RLPs. A recent study reported that combined GPIHBP1- and LDLR-deficiency results in more extensive and advanced atherosclerosis as well as rare lesions in coronary arteries in diabetic mice, as compared to *Ldlr*^−/−^ controls (107). Therefore, the combination of severe hypertriglyceridemia, LDLR-deficiency and diabetes appears to worsen the effects of hypertriglyceridemia on atherogenesis.

Data obtained from studies of GPIHBP1-deficient mice support the proposal that large TRLs can be atherogenic but are much less atherogenic than RLPs and LDL.

#### Combined Lipase-Deficiency (Lipase Maturation Factor 1)

Combined lipase deficiency (cld) was identified in a spontaneous mouse mutant deficient in both LPL and hepatic lipase (HL) activity (108). These mice display postpartum chylomicronemia and death after nursing (108, 109). Although LPL protein is normally expressed in cld mutant mice, it fails to attain the catalytically active conformation and is subject to retention in the ER and subsequent degradation (110). The cld mutation was mapped to the *Lmf1* (lipase maturation factor 1; formerly known as *Tmem112*) gene in murine chromosome 17. Subsequent studies showed that LMF1 localizes to the ER membrane and facilitates maturation of LPL, HL, and endothelial lipase (EL) (109).

Overexpression of *LMF1* in adipose tissue, heart, and muscle tissues resulted in increased LPL activity in all of these tissues (111). Other naturally occurring murine mutations in Lmf1 have also been demonstrated to compromise LMF1 function *in vitro* (112). The hypertriglyceridemia in cld mice suggested that the human ortholog LMF1 on chromosome 16 could be a candidate gene for regulating TG metabolism, and various *LMF1* mutations have been identified in patients with hypertriglyceridemia (113). Thus, a role for LMF1 in regulating TG metabolism is indicated by phenotypic and functional consequences of rare large-effect mutations in humans and mice. However, its common variants interrogated using GWAS failed to produce an association signal with hypertriglyceridemia (114).

The effect of LMF1 on atherosclerosis in mouse models is unknown. One would predict that LMF1 loss-of-function mutations would have effects on atherosclerosis surpassing those of LPL-deficiency because LMF1 is also needed for maturation of HL and EL.

#### Apolipoprotein C2 (APOC2)-Deficiency

APOC2 is a crucial cofactor for LPL activity. Mechanistically, APOC2 binds LPL and has been proposed to promote its activation by helping guide the TG substrate into the active site of LPL (115, 116). APOC2 is primarily expressed by the liver and secreted into plasma, but it is also produced at lower levels by other tissues, like the intestine, macrophages, adipose tissue, brain, skin, lungs, retina, and retinal pigment epithelium (115). Like other APOC apolipoproteins, APOC2 moves from APOB-containing lipoproteins to HDL during lipolysis (117, 118). In humans, *APOC2* loss-of-function mutations can promote a prodigious rise in plasma TG levels due to chylomicronemia and consequently can provoke acute pancreatitis (119). Likewise, mice expressing a homozygous loss-of-function *Apoc2* mutant (120) have increased plasma TGs (~700 mg/dL) and low HDL-C (~30 mg/dL) compared with wildtype mice (TGs; 60 mg/dL; HDL-C, 60 mg/dL), consistent with its role as an LPL cofactor (Figure 2).

Whether the effect of APOC2-deficiency on atherosclerosis phenocopies the effect of LPL-deficiency in mice needs further investigation.

#### Apolipoprotein C3 (APOC3) Overexpression

Human studies have firmly linked APOC3 to plasma TG levels (121–123). APOC3 is mainly produced by the liver and to a lesser extent, by the intestine. Like APOC2, it shuttles between APOB-containing lipoproteins and HDL (124, 125). Thus, while APOC3 is associated with chylomicrons, VLDL, and their RLPs, lipolysis of these particles is associated with transfer of APOC3 to HDL. Some APOC3 is also associated with LDL. APOC3 prevents TRL and RLP clearance by at least two different mechanisms: (i) it inhibits the action of LPL through a mechanism believed to be due to blocking of LPL's access to its triacylglycerol substrates when LPL is bound to GPIHBP1 (126); and (ii) it interferes with the binding of APOE to hepatic receptors of the LDL receptor family, thus resulting in a delayed catabolism and clearance of TRLs and RLPs (67). Early studies showed that at high concentrations, APOC3 also inhibits HL, thus further reducing lipolysis (127). However, no changes in HL activity were observed in a recent study of mice treated with an APOC3 antisense oligonucleotide (ASO) (128), suggesting that HL inhibition is not a significant mechanism of APOC3 *in vivo*. Overexpression of APOC3 in mice leads to increased TG levels because of the low LPL catalytic activity and delayed catabolism of VLDL and chylomicrons (129, 130).

When mice carrying the human APOC3 transgene were crossed with *Ldlr*^−/−^ mice, significant increases in VLDL and RLPs were observed concomitant with increased atherosclerosis, as compared with *Ldlr*^−/−^ controls (46). Later studies confirmed that APOC3 overexpression promotes restenosis and atherosclerosis (47, 131).

Together, there is strong evidence to suggest that APOC3 overexpression promotes atherosclerosis in mouse models. This effect is likely due, at least in part, to increased retention of RLPs in the artery wall due to the slower clearance rate of RLPs. Whether APOC3 also has direct effects on lesional cells independent of its effects on TRL and RLP catabolism, as has been suggested (47, 132–134), requires further study.

#### Apolipoprotein A5 (APOA5)-Deficiency

The *APOA5* gene is located in the *APOA1/APOC3/APOA4/APOA5* gene cluster on chromosome 11q23 in humans, a locus well-known to play a major role in regulating plasma cholesterol and TG levels (135). APOA5 is produced by the liver and circulates in plasma at very low concentrations (0.1–0.4 mg/mL). Mice lacking APOA5 exhibit TG levels in the 800 mg/dL range (136). In mice with hepatic APOA5 downregulation using an ASO, plasma TG levels were increased modestly (to ~150 mg/dL) associated with reduced LPL activity (137). It has been suggested that the mechanism whereby APOA5 increases LPL activity (135) is by guiding VLDL and chylomicrons to endothelial cell-bound LPL for lipolysis (138). Consistently, metabolic studies *in vivo* show that APOA5 enhances the catabolism of TRL rather than reducing TRL production.

The atherosclerosis phenotype of *Apoa5*^−/−^ mice has not been reported. However, consistent with the increased TRL/RLP clearance by APOA5 (139), overexpression of human *APOA5* in *Apoe*^−/−^ mice resulted in decreased atherosclerotic lesion formation (48).

Together, these results show that APOA5 plays a critical role in promoting TRL and RLP clearance, an effect associated with protection from atherosclerosis.

#### Cyclic AMP-Responsive Element-Binding Protein H (CREB-H)-Deficiency

The transcription factor CREB-H (*CREB3L3*) is required for the maintenance of normal plasma TG concentrations, and loss-of-function mutations in the *CREB3L3* gene are associated with severe hypertriglyceridemia in humans (140). CREB-H most likely works through indirectly increasing LPL activation, because its target genes include APOC2 and APOA5, although it also regulates other genes of importance in cardiometabolic diseases (140). CREB-H deficiency in *Ldlr*^−/−^ mice leads to increased VLDL-TG levels and increased atherosclerosis (49).

### Mouse Population Models and Models of Secondary Hypertriglyceridemia

The genetic mouse models of hypertriglyceridemia described above are primarily based on monogenic alterations on a single genetic background. The significant contributions of complex traits and polygenic contributions to hypertriglyceridemia in humans (141, 142) are therefore difficult to study. To tackle this issue, a panel of over 100 inbred strains of mice, known as the Hybrid Mouse Diversity Panel was generated by the Lusis laboratory (143). This panel offers important insights into genetically-derived differences in phenotype among inbred mice, and has been used to show that plasma TGs in mice fed a high fat/high sucrose diet are regulated by different quantitative trait loci (QTLs) on mouse chromosome 7 in male and female mice (144). Mice made hyperlipidemic by expression of human APOE-Leiden and human cholesteryl ester transfer protein (CETP) revealed that plasma TG levels varied widely in the different genetic backgrounds (from ~100 to 1,500 mg/dL) and highlighted a TG locus on chromosome 1, containing several genes with unknown functions in TG metabolism (145). Plasma TG levels were not associated with atherosclerosis in these studies. It would be interesting to perform similar studies to identify loci associated with elevated RLP levels to test the hypothesis that RLPs, rather than TGs, confer pro-atherogenic effects.

Other mouse models have been used to study the effects of non-genetic conditions that elevate plasma TG levels (referred to as secondary hypertriglyceridemia in humans). For example, mice made diabetic using the beta cell toxin streptozotocin or a virus develop elevated plasma TG levels. Interestingly, in a virally-induced diabetes model (a model of poorly controlled type 1 diabetes), plasma TG levels are elevated without a significant increase in plasma APOB (which is most abundant in LDL), suggesting a selective effect on TRLs (45). The mechanisms behind the elevated TG levels include reduced LPL activity (146) and increased APOC3 levels (45), leading to reduced TRL and RLP clearance. Furthermore, intensive insulin therapy but not blood glucose lowering by a sodium-glucose cotransporter 2 inhibitor was shown to prevent the effects of diabetes on APOC3 elevation (45). The accelerated atherosclerosis in diabetic mice was prevented by APOC3 silencing as discussed below. Thus, elevated APOC3 appears to explain the effect of diabetes on TGs and atherosclerosis in this model. Mouse models of type 2 diabetes can also exhibit elevated plasma TG levels. For example, in diabetic KKA^y^ mice (a model of polygenic diabetes) elevated plasma TG levels have been shown to be due to reduced hepatic TRL clearance through syndecan 1 and flotillin 1-mediated endocytosis (147). Together, these studies suggest that the principal cause of the elevated TG levels in diabetes models is ineffective TRL clearance rather than increased hepatic VLDL production (146).

### Rat Models

The rat was used for some of the early studies on TRL and RLP metabolism. For example, studies in hepatectomized rats demonstrated the importance of the liver in RLP removal (148) and studies in rats injected with chylomicrons demonstrated that phospholipids and some apolipoproteins are transferred from chylomicrons to HDL during lipolysis (149).

There are also rat models of hypertriglyceridemia. Rats homozygous for the corpulent gene (cp/cp) become obese, insulin resistant, and hypertriglyceridemic, primarily due to hepatic hypersecretion of VLDL (150). The cp trait results from a premature stop codon in the extracellular domain of the leptin receptor and therefore these rats lack a functional leptin receptor (151). Among the different cp strains, the JCR:LA-cp rat strain displays hyperlipidemia associated with APOB48-containing lipoprotein particles, particularly in the early post-prandial phase (152), and vasculopathy with atherosclerotic lesions and associated ischemic myocardial lesions (54), consistent with a pro-atherogenic effect of VLDL or its lipolysis products. However, other mechanisms, including leptin-related effects could have contributed to the atherosclerosis phenotype in this model.

Different models of APOE-deficiency have been generated by various gene editing strategies in rats. These *Apoe*^−/−^ rats display a range of atherosclerosis phenotypes, ranging from no detectable lesions, to very early signs of lipid deposition and atherosclerosis (55, 56, 153, 154), at least as compared with *Apoe*^−/−^ mice, to more advanced lesions (57). Thus, one *Apoe*^−/−^ rat model exhibits an atherosclerosis phenotype similar to that of the *Apoe*^−/−^ mice described above but lesion progression appears to be generally slower than that in the mouse. The reason for the differences in atherosclerosis burden in the different *Apoe*^−/−^ rat models is so far unclear.

Finally, overfeeding rats with sucrose or fructose induces hypertriglyceridemia. Whereas sucrose favors hepatic fatty acid esterification and VLDL synthesis, fructose-rich diets deteriorate VLDL-TG catabolism (155). Wistar rats fed a high-sucrose (68%) or fructose (60%) diet have a 4-fold increase in TG levels after 3 and 8 weeks, respectively (155, 156).

So far, studies in rats support the more detailed mechanistic studies on TRLs and atherosclerosis in mouse models. Certain characteristics of lipid metabolism in rats are closer to those of humans as compared to mice (157), hence development of rat atherosclerosis models might provide advantages over mouse models. In addition, rats, due to their size have the benefit of easier blood collection, dissection of blood vessels, and other tissues, and thus are beneficial for atherosclerosis studies. Further analysis of different rat models and the role of TRLs and RLPs in atherosclerosis is needed as technologies for gene manipulation in rats are becoming more widely used.

### Rabbit Models

Lipoprotein metabolism of rabbits resembles that of humans in composition of APOB-containing lipoproteins (158), production of APOB100-containing VLDL by the liver (159), presence of plasma CETP (which is lacking in mice and rats) (160), and high absorption rate of dietary cholesterol (161). One difference is the relatively low expression of HL in rabbits (162). Watanabe Heritable Hyperlipidemic (WHHL) rabbits with a natural defect in LDLR function, when fed high-fat diet, exhibit elevated plasma lipid levels and aortic lesions within 8 weeks. At 16 weeks, the lesions are advanced, with lipid core formation and calcification (58, 59). Selective crossing of WHHL rabbits with Japanese White (JW) rabbits and screening for blood TG levels led to the identification of two hypertriglyceridemic strains (163). The high hereditary hypertriglyceridemia (TGH) rabbits show autosomal recessive inheritance, and exhibit TG values >500 mg/dL. In addition, physical examination and necropsy of TGH rabbits revealed xanthomas and lesions of aortic atherosclerosis similar to those in human disease (163). A cross-breed between TGH rabbits and JW rabbits resulted in a post-prandial hypertriglyceridemic rabbit model (PHT) that exhibits remarkably high levels of postprandial TG (500–3,000 mg/dL) (163, 164) and increased atherosclerosis (60). In addition, the St. Thomas Hospital (STH) rabbit displays a Mendelian form of hypertriglyceridemia accompanied by increased APOB lipoprotein production and accelerated atherosclerosis (61).

The rabbit models described above confer advantages over mice and rats, but mechanistic studies on the role of TRLs in atherosclerosis lag behind those of the mouse models.

### Pig Models

Lipid metabolism and cardiovascular physiology in pigs share several similarities with humans, and pigs develop atherosclerosis and a human-like lipoprotein profile without genetic manipulation. Hence, some pig models of hypertriglyceridemia have been developed. Göttingen minipigs under a dietary intervention consisting of a high-fat/high-energy diet have significantly elevated plasma TG levels (62) and develop atherosclerosis. Transgenic pigs expressing human APOC3 in the liver and intestine have also been developed, and these animals have increased plasma TG levels (2.5-fold), but normal total cholesterol and HDL-C levels. They also exhibit delayed TG absorbance and clearance (165), consistent with the known role of APOC3 in impeding TRL catabolism.

### Zebrafish Model

The zebrafish is an emerging animal model for the study of abnormalities of lipid metabolism and associated diseases. In 2015, zebrafish expressing a non-functional APOC2 mutant were developed (63). These mutant zebrafish displayed decreased plasma TG lipase activity and severe hypertriglyceridemia, mainly due to buildup of chylomicrons. The hypertriglyceridemia was then rescued by injection of plasma from wildtype zebrafish with functional APOC2 or by injection of a human APOC2 mimetic peptide. The zebrafish mutants accumulated lipid and lipid-laden macrophages in the vasculature even on a normal diet—early events in atherosclerosis progression. However, fish do not express all genes contributing to hypertriglyceridemia in humans. For example, GPIHBP1 is not expressed in fish. It is believed that GPIHBP1 originated in eutherian mammals from a gene duplication event of an ancestral LY6-like gene (166). Nevertheless, the zebrafish model could be used to test factors affecting hypertriglyceridemia in a time and cost-effective manner.

### Non-human Primate Models

Rhesus macaques, when fed a high-fructose diet, develop hypertriglyceridemia along with many other features of the metabolic syndrome, including central obesity, dyslipidemia, and inflammation observed in humans. In these insulin resistant monkeys, fasting TG levels were increased by 60% at 6 months and by almost 90% at 12 months (167). Fish oil supplementation reduced hypertriglyceridemia in this model of fructose-fed rhesus monkeys (168). In another study aimed at testing the effect of APOC3 inhibition using an ASO, rhesus monkeys were made hypertriglyceridemic via administration of a high-fructose supplement for 16 weeks and were then treated with APOC3 ASO for 12 weeks as the high-fructose diet was maintained (169). With fructose supplementation, plasma TG levels were increased by at least 3-fold over initial baseline levels in all treatment groups (40 mg/dL at day 1 vs. 140 mg/dL at day 112), just before initiation of dosing. APOC3 ASO significantly reduced fasting TG levels and postprandial TGs, as compared with monkeys receiving control ASO. Since non-human primates are more closely related and have similar lipid profiles to humans (170), the fructose-fed rhesus monkeys can represent a translatable model for studying the effects of TGs and novel TG-lowering strategies on atherosclerosis in humans.

## Emerging Strategies to Lower Hypertriglyceridemia and Their Effects on Atherosclerosis in Animal Models

### APOC3 Inhibition

The physiological role of APOC3 is likely to ensure delivery of fatty acids derived from TGs to adipose tissue, muscle and the heart both after a meal and between meals by slowing the clearance of TRLs and RLPs. Because human studies convincingly show that loss-of-function mutations in *APOC3* are associated with lower TG levels and cardioprotection (121, 122, 171–174), APOC3 has recently emerged as a drug target for the treatment of hypertriglyceridemia and possibly the associated increase in CVD risk (18, 175–177).

The human studies spurred pharmaceutical development of APOC3 inhibitors in the form of ASOs, interference RNAs and monoclonal antibodies (122, 168, 178). Volanesorsen (IONIS-APOCIII Rx) is a second-generation ASO drug targeted to *APOC3* mRNA, which effectively reduces plasma TG levels in human clinical trials (175–177). However, this ASO was not approved by the FDA for the treatment of familial chylomicronemia syndrome due to adverse effects related to thrombocytopenia. The Committee for Medicinal Products for Human Use of the European Medicines Agency however, authorized conditional marketing for Waylivra (volanesorsen) as an adjunct to diet in adult patients with genetically confirmed familial chylomicronemia syndrome who are at high risk for pancreatitis, in whom response to diet and TG-lowering therapy has not worked (123). New formulations of APOC3 inhibitory drugs are likely to reduce side-effects. CVD outcomes studies have not yet been performed in humans using APOC3-lowering therapies.

Animal models have provided additional mechanistic insight into the protective effects of APOC3 inhibition. APOC3 ASOs similar to volanesorsen have been effective in reducing APOC3 expression and plasma TGs in several rodent models and in non-human primates (45, 169). Mechanistic studies in mice using the APOC3 ASO showed that plasma TG levels were lowered by increasing activation of LPL as well as furthering the clearance of TRLs by the liver, via the LDLR family of receptors (67). Importantly, inhibition of APOC3 lowers TRLs without significantly affecting LDL levels or APOB100 levels in both humans and mice (45, 174).

A recent study analyzing both human samples and a mouse model showed that elevated serum levels of APOC3 predict coronary artery disease events in human subjects with type 1 diabetes independent of LDL-C and several other risk factors, and that plasma levels of APOC3 were significantly increased in a mouse model of type 1 diabetes (45). There was a striking protective effect of APOC3 inhibition by ASO on both early and more advanced atherosclerosis in this mouse model of type 1 diabetes-accelerated atherosclerosis (45). This effect was associated with protection from accumulation of APOB, APOE, and APOC3 in the artery wall (most likely associated with reduced RLPs retention) as well as protection from cholesteryl ester accumulation in macrophages from diabetic mice.

Together, these studies strongly suggest that APOC3 inhibition could be effective in treating hypertriglyceridemia and preventing atherosclerotic CVD, especially in conditions in which APOC3 levels are increased.

### APOC2 Mimetic Peptide

As discussed above, APOC2 is an essential cofactor for LPL activation and APOC2 therefore promotes TRL hydrolysis. Current treatment options for severely hypertriglyceridemic patients with loss-of-function mutations in *APOC2* include a stringent low-fat diet and plasma exchange with APOC2-containing donor plasma as a direct and lifesaving procedure during severe pancreatitis episodes (179). Recently, APOC2 mimetic peptides have been developed for treatment of hypertriglyceridemia, especially in APOC2-deficient patients (115). These peptides, which incorporate the third helix of APOC2, are promising as potential future therapy. Intravenous injection of one such peptide normalized TGs in a mouse model of APOC2-deficiency (120). Furthermore, the APOC2 mimetic peptide potentiated LPL activity in other non-APOC2-deficient hypertriglyceridemic conditions, like APOE-deficiency (180).

The most potent peptide (D6PV), developed on the basis of structural insights gained from all-atom molecular dynamics simulations, directly activates LPL and also inhibits APOC3's action. D6PV treatment drastically lowers TGs in APOC2-deficient mice and in human APOC3-transgenic mice (181). This peptide shows some LPL-independent TG lowering activity, probably because it has dual APOC2 and APOC3 antagonistic effects, as it partially decreases plasma TG in whole-body inducible *Lpl*^−/−^ mice. It also lowers TG levels in non-human primates (181). Its effect on atherosclerosis in animal models (and humans) is so far unknown.

### Angiopoietin-Like 3 (ANGPTL3) Inhibition

ANGPTL3 is a hepatically secreted protein, which inhibits the vascular lipases LPL and EL. Loss-of-function mutations in *ANGPTL3* lead to familial combined hypolipidemia, characterized by low circulating levels of LDL-C, HDL-C, and TGs (182) and apparent protection against atherosclerotic CVD (182). ANGPTL3 acts to increase TG levels by catalyzing the irreversible unfolding of LPL's hydrolase domain, resulting in a loss of both TG hydrolase activity and esterase activity (104). A complex consisting of ANGPTL3 and ANGPTL8 (another protein in the ANGPTL family) has a markedly increased ability to inhibit LPL activity, likely by increasing the binding of ANGPTL3 to LPL (183).

The favorable lipid effects seen in animal models and humans with loss of ANGPTL3 function have led to a spurt in development of ANGPTL3 inhibitors. Evinacumab is a monoclonal antibody, which binds to ANGPTL3 and completely reverses its inhibitory activity on LPL and EL (184). *In vivo* studies on normolipidemic mice showed a dose-dependent reduction in TG, total cholesterol, LDL-C and HDL-C serum levels after subcutaneous injections of evinacumab. An increase in LPL and EL activity has been recorded in normolipidemic wildtype mice as well as in dyslipidemic wildtype (C57BL/6) mice, in db/db mice and in APOE^*^3-Leiden mice in which ANGPTL3 is inhibited (184). The same results have been obtained by treating dyslipidemic cynomolgus monkeys (185). Mice on evinacumab treatment showed a significant decrease in total cholesterol and TG levels as well as in atherosclerotic lesion size. ASO-mediated ANGPTL3 inhibition in mice with different lipid phenotypes (wildtype C57BL/6 mice, *Ldlr*^−/−^ mice, *Apoc*3^−/−^ *Ldlr*^−/−^ mice, heterozygous *Apoc3*^+/−^ *Ldlr*^−/−^ mice, diet-induced obese mice, and mice over-expressing human APOC3) resulted in lowered plasma TGs, LDL-C and HDL-C and reduced atherosclerosis (50). Another group targeted ANGPTL3 using a modified CRISPR-Cas9 platform. Wildtype mice and hyperlipidemic *Ldlr*^−/−^ mice targeted with this ANGPTL3 silencing strategy showed reduced plasma TG and total cholesterol levels (186).

Together, these findings suggest that ANGPTL3 is a promising target for TG lowering and perhaps prevention of atherosclerotic CVD.

### Angiopoietin-Like 4 (ANGPTL4) Inhibition

ANGPTL4 was identified as a fasting-induced adipose factor and was found to act as a potent LPL inhibitor (187–189). ANGPTL4 is more widely expressed than ANGPTL3, and is highly expressed in the liver, adipose tissue, hematopoietic cells, and other tissues. Studies in ANGPTL4 transgenic mice and in mice with adenoviral ANGPTL4 overexpression show decreased LPL activity, delayed TG clearance, and increased plasma TG levels (190, 191). The increase in plasma TGs and free fatty acids associated with ANGPTL4 overexpression is thought to be independent of food intake and hepatic VLDL secretion. Instead, ANGPTL4 has recently been shown to inactivate LPL by catalyzing LPL unfolding (14). Conversely, ANGPTL4-deficient mice exhibit increased plasma LPL activity, increased TG clearance, decreased plasma TG levels, and reduced atherosclerosis (51, 190). Several GWAS in humans corroborate the findings of ANGPTL4-mediated regulation of lipid metabolism in mice (192, 193). As a result, ANGPTL4 was targeted for TG lowering using monoclonal antibodies. Humanized mice treated with ANGPTL4 blocking antibodies display reduced plasma TG levels (194). Moreover, cynomolgus monkeys and hyperlipidemic rhesus monkeys also display reduced TGs when treated with ANGPTL4-neutralizing antibodies (194). However, mice lacking ANGPTL4, when fed a high-fat diet, demonstrate a severe inflammatory phenotype and increased mortality (195).

Although ANGPTL4 inhibition is effective in lowering TGs in mice and primates and also reduces atherosclerosis progression, an important question remains regarding the safety of this approach due to the mesenteric lymphadenopathy seen in high fat-fed animals in which ANGPTL4 is blocked.

## Summary

We have reviewed the most common animal models of hypertriglyceridemia and the critical mechanistic insights provided by these models. One important conclusion is that these models, in general, are consistent with the proposal that RLPs derived from TRLs are highly atherogenic whereas hypertriglyceridemia due to accumulation of very large TRLs is not markedly atherogenic in the absence of TRL lipolysis products. Combination of mechanistic animal studies and studies on isolated lesional cells indicate that RLPs are more atherogenic than large TRLs, perhaps because they more readily enter the artery wall, and because they are enriched in cholesterol relative to TGs, which promotes pro-atherogenic effects in lesional cells. Animal studies have also revealed that RLPs are cleared through hepatic LDLR and LRP1 receptors, and that LPL has effects additional to its role in TG hydrolysis in capillaries, including by acting locally in the lesion. However, definite proof that RLPs rather than nascent TRLs are the cause of greater CVD in patients with hypertriglyceridemia and/or defects in genes leading to reduced LPL actions is still missing and will require further research.

## Future Directions

Elevated plasma TG concentrations appear to be a predictor of CVD risk in humans. GWAS have greatly widened the repertoire of TG-associated genes linked to CVD risk. Clinical hypertriglyceridemia has a complex genetic basis that includes a burden of both common variants, such that each has a relatively small effect on TG levels, and rare variants, such that each has a relatively large effect on TG levels. It is quite clear that LPL and factors affecting its activity or function play a major role in atherogenesis, at least in animal models.

The multifactorial nature of hypertriglyceridemia makes it challenging to draw clear conclusions concerning its atherogenicity. A more complete understanding of the genes and variants that modulate plasma TGs requires strong functional validation at all stages: *in vitro, in vivo*, and ultimately in clinical trials, and will be crucial for identifying key players in TG metabolism, which might help specifying new directions for therapeutic interventions. Many such studies are ongoing. For instance, studies of the effect of hypertriglyceridemia in the context of atherosclerosis regression in animal models, in which the confounding factor of hypercholesterolemia is absent, are likely to provide important insights. Furthermore, studies of the role of RLPs in CVD associated with diabetes or other conditions associated with increased CVD risk will be important. Additionally, the role of LPL in peripheral TG metabolism vs. its effect locally in the artery wall and on macrophage lipid metabolism can be dissected using newer genetic models. Moreover, the mechanistic role of how fish oils (in particular eicosapentaenoic acid) protect against CVD risk, and whether this protection is related to TG lowering or other mechanisms should be assessed in animal models of hypertriglyceridemia. New tools and methods to measure and evaluate TRLs and RLPs will also be critical for understanding if TRLs and RLPs have distinct effects on atherosclerotic lesions and as risk factors for CVD (23).

The last decade saw the discovery of new drug targets and the early phase clinical trials testing these targets, including APOC3 and ANGPTL3, which both inhibit LPL activity. The development of several different strategies for reducing TG levels is likely to be more specific and effective than drugs currently on the market. Animal models will help to provide mechanistic insight through reverse translation of clinical observations. There is hope that the current decade will reveal whether such strategies will also prevent CVD risk in patients with hypertriglyceridemia and others with impaired TRL and RLP catabolism.

## Author Contributions

DB wrote the manuscript. KB wrote parts of the manuscript and edited the full manuscript. All authors contributed to the article and approved the submitted version.

## Conflict of Interest

The authors declare that the research was conducted in the absence of any commercial or financial relationships that could be construed as a potential conflict of interest.
